# Efficacy of Mobile Instant Messaging–Delivered Brief Motivational Interviewing for Parents to Promote Physical Activity in Pediatric Cancer Survivors: A Randomized Clinical Trial

**DOI:** 10.1001/jamanetworkopen.2022.14600

**Published:** 2022-06-14

**Authors:** Ankie Tan Cheung, William Ho Cheung Li, Laurie Long Kwan Ho, Godfrey Chi-Fung Chan, Huen Sum Lam, Joyce Oi Kwan Chung

**Affiliations:** 1 The Nethersole School of Nursing, The Chinese University of Hong Kong, Sha Tin, Hong Kong; 2 Department of Paediatrics and Adolescent Medicine, Li Ka Shing Faculty of Medicine, The University of Hong Kong, Hong Kong; 3 Department of Paediatrics and Adolescent Medicine, Hong Kong Children's Hospital, Hong Kong Children’s Hospital, Hong Kong; 4 Hong Kong Council for Accreditation of Academic and Vocational Qualifications, Hong Kong; 5 School of Nursing, The Hong Kong Polytechnic University, Hong Kong

## Abstract

**Importance:**

Physical activity has beneficial effects that mitigate cancer- and treatment-related late effects. However, children who survive cancer are often physically inactive. Brief motivational interviewing may be an effective approach for increasing children's physical activity levels.

**Objective:**

To examine the effects of mobile instant messaging–delivered brief motivational interviewing for parents in promoting regular physical activity in children who have survived cancer.

**Design, Setting, and Participants:**

An assessor-blinded randomized clinical trial was conducted at 2 Hong Kong pediatric oncology outpatient clinics from March 1, 2019, to January 29, 2021. A total of 161 children who had survived cancer, aged 9 to 16 years, and their parents were randomized (1:1) to an intervention or control group.

**Interventions:**

The intervention group received a 6-month mobile instant messaging–delivered brief motivational interviewing using a strategy menu. Parent-child dyads in both groups received a health advice session and were directed to a physical activity website at baseline.

**Main Outcomes and Measures:**

The primary outcome was the children’s physical activity levels at 12-month follow-up, measured by the Chinese University of Hong Kong: Physical Activity Rating for Children and Youth (total sores: 0-10, higher scores indicate greater physical activity levels). Secondary outcomes were cancer-related fatigue levels, handgrip strength, peak expiratory flow rate, and quality of life. Intention-to-treat analysis was performed.

**Results:**

Of the 161 children included in the study, 93 were boys (57.8%), and the mean (SD) age was 12.4 (2.4) years. Generalized estimating equation analyses showed a significant improvement in outcomes in the intervention group compared with the control group: physical activity levels (group-by-time interaction, 6 months: β = 3.09; 95% CI, 2.65-3.53; *P* < .001; 12 months: β = 3.91; 95% CI, 3.45-4.36; *P* < .001), cancer-related fatigue (6 months: β = −5.69; 95% CI, −8.03 to −3.35; *P* < .001; 12 months: β = −9.16; 95% CI, −11.31 to −7.00; *P* < .001), left handgrip strength (6 months: β = 2.69; 95% CI, 0.96-4.43; *P* = .002; 12 months: β = 5.52; 95% CI, 3.70-7.33; *P* < .001), right handgrip strength (6 months: β = 2.75; 95% CI, 1.01-4.50; *P* = .002; 12 months: β = 5.45; 95% CI, 3.62-7.27; *P* < .001), peak expiratory flow rate (12 months: β = 28.51; 95% CI, 16.10-40.92; *P* < .001), and quality of life (6 months: β = 5.01; 95% CI, 1.19-8.82; *P* = .01); 12 months: β = 14.19; 95% CI, 10.84-17.54; *P* < .001).

**Conclusions and Relevance:**

In this randomized clinical trial, mobile instant messaging–delivered brief motivational interviewing was effective in promoting the adoption and maintenance of regular physical activity and ameliorating cancer- or treatment-related adverse effects in children who survived cancer.

**Trial Registration:**

ClinicalTrials.gov Identifier: NCT03859271

## Key Points

**Question:**

Can brief motivational interviewing delivered via mobile instant messaging promote regular physical activity in children who have survived cancer?

**Findings:**

In this randomized clinical trial involving 161 children who survived cancer and their caregivers, brief motivational interviewing delivered via instant messaging significantly increased physical activity levels, reduced cancer-related fatigue, and improved handgrip strength and quality of life during a 12-month study period. Moderate to vigorous physical activity levels in intervention participants increased by 72.8%, compared with 6.3% in controls.

**Meaning:**

The intervention used in this randomized clinical trial was effective in promoting regular physical activity in children who survived cancer; further investigation is warranted to enhance long-term sustainability of the intervention effect.

## Teaser

This randomized clinical trial examines the use of motivational interviewing delivered to parents of pediatric cancer survivors through mobile text messaging on physical activity levels in their children.

## Introduction

The average 5-year survival rate for most types of pediatric cancers is approximately 85% owing to advances in cancer treatments.^1^ Despite this survival rate, the high rates of toxic effects and low specificity of cancer treatment given at an early age induces many late effects that have a detrimental effect on survivors’ physical and psychosocial health.^2,3,4,5^ A cohort study of 10 397 children who survived cancer reported that an estimated two-thirds of survivors developed at least 1 late effect.^4^ Common late effects reported by these children included persistent cancer-related fatigue,^6^ reduced functional capacity,^7^ and decreased muscle strength and endurance.^8^ These effects substantially disrupt the children’s daily functioning and quality of life (QOL).^6^ A high prevalence of obesity has been reported among children who survive cancer in Western countries, which further increases the risk of morbidity and mortality.^9,10,11^

Physical activity (PA) is beneficial in preventing and attenuating many adverse late effects following pediatric cancer and treatment.^12,13,14^ However, most children who survive cancer do not participate in sufficient PA to obtain these health benefits.^15,16,17^ Physical inactivity in children who survive cancer is of emerging concern because it may compound late effects and reduce the functional capacity of many organ systems, which together can lead to poor health and premature death.^18^ Studies have found that approximately 92% of Hong Kong Chinese children who survive cancer and 52% of their Western counterparts did not adhere to the minimum PA guideline of 60 minutes of moderate to vigorous PA per day recommended by the World Health Organization.^15,16,17^

Parents play an important role in promoting their child’s PA behavior via support and role modeling.^19,20,21,22^ However, many Chinese parents have been reported to lack the motivation to encourage their children to perform regular PA owing to their unawareness of the importance and health benefits associated with PA in ameliorating the late effects of cancer and its treatment.^15,23^ In addition, Chinese parents placed a strong emphasis on their child’s academic achievement over PA owing to the extremely competitive environment for education in Hong Kong.^24,25^ Therefore, it is essential to involve parents in PA interventions to motivate them to encourage their child in adopting and maintaining regular PA throughout their survivorship.

**Figure 1.**
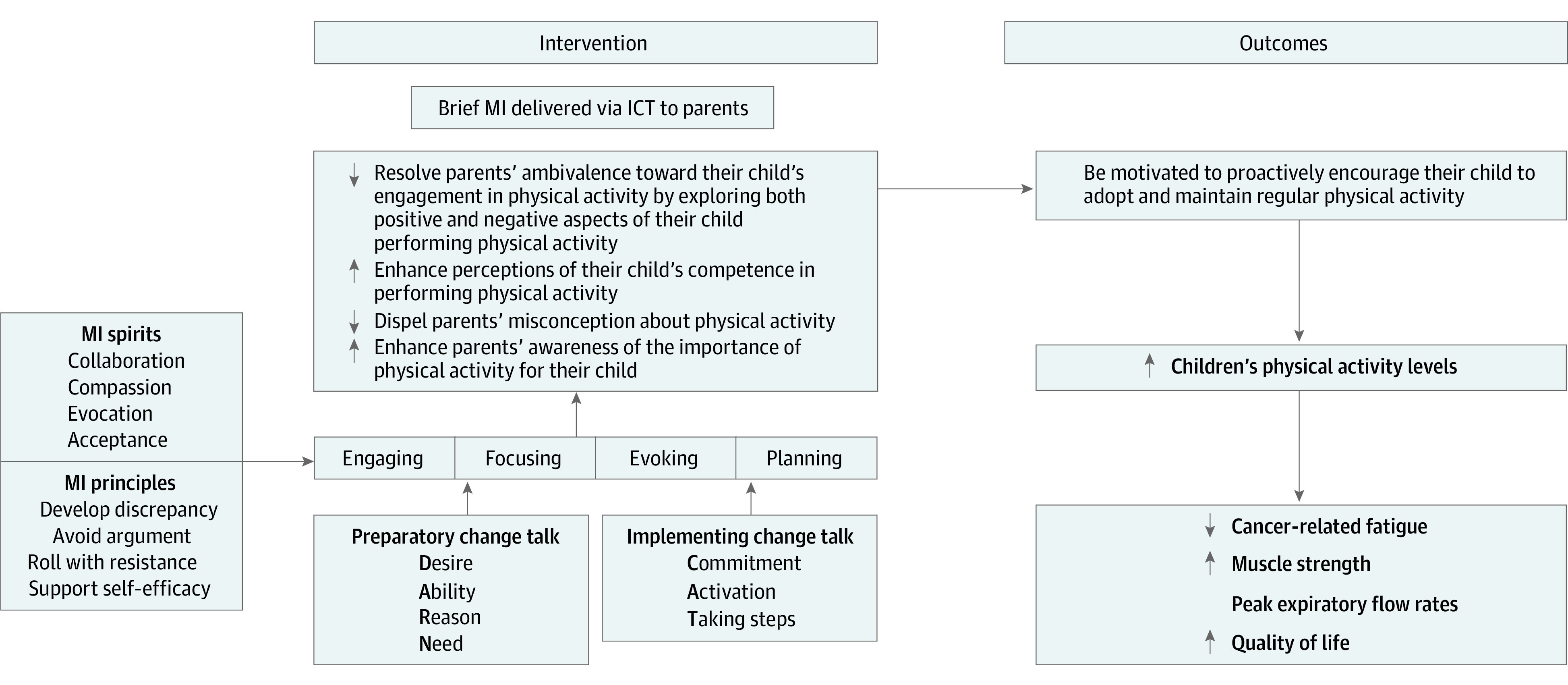
Theoretical Framework of the Brief Motivational Interviewing Attentive to the motivational intervention (MI) spirit of collaboration, compassion, evocation, and acceptance, and the principles of expressing empathy, highlighting discrepancies, avoiding arguments, rolling with resistance, and supporting self-efficacy, this study explored the potential to enhance parents' intrinsic motivation to encourage their child to engage in physical activity (PA) through an MI attuned to their perspectives and ambivalence. The reasoning, in other words, was that the brief MI sessions would support and enhance parents' autonomy and self-efficacy and convince them to motivate their children to be more physically active. Throughout the 4 fundamental processes of brief MI with the adaptation of the menu of strategies and use of micro skills (eliciting and reinforcing preparatory/implementing change talk), it is anticipated that parents' ambivalence toward the engagement in PA for their child will be resolved by exploring both positive and negative aspects of ambivalence (ie, pros and cons of their child performing PA) and their discrepancy in changing the behavior will be developed (indicated by preparatory and implementing change talks). Brief MI could also enhance parents' perceived competency in PA by providing resources and information regarding PA in children who have survived cancer. This probably enables the parents to act as role models in PA behavior and hence proactively motivate their child to adopt and maintain regular PA. The expectation was that PA would improve the children's cancer-related fatigue levels, muscle strength, lung function, and quality of life.

Research found that educational interventions alone are inadequate to initiate changes and promote adherence to healthy behavior.^26,27^ Evidence suggests the benefits of using motivational interviewing (MI) with parents to promote health-enhancing behavior in their children.^28,29^ Motivational interviewing is a client-centered directive counseling technique that helps clients resolve ambivalence and enhance the intrinsic motivation to elicit changes in behavior.^30,31,32^ Figure 1 shows the theoretical framework of the brief MI intervention.

Mobile instant messaging applications are widely used in different fields owing to their low cost, high flexibility, and feasibility.^33^ Therefore, we used this technology to deliver brief MI via WhatsApp or WeChat to parents of children who have survived cancer. Although the benefits of brief MI in promoting health behavior changes have been consistently reported in various populations,^28,29^ few studies have examined its use in promoting PA among children who have survived cancer. Thus, we conducted a randomized clinical trial to examine the effects of a brief MI delivered to parents via mobile instant messaging applications on PA levels, fatigue, muscle strength, peak expiratory flow rate (PEFR), and QOL in Chinese children who have survived cancer.

## Methods

### Study Design

This was an assessor-blinded, multicenter randomized clinical trial conducted at 2 pediatric oncology outpatient clinics in 2 public hospitals in Hong Kong. This study followed the Consolidated Standards of Reporting Trials (CONSORT) reporting guideline. Ethical approval was granted by the institutional review board of the University of Hong Kong/Hospital Authority Hong Kong West Cluster and Hong Kong Children’s Hospital. Parents provided written informed consent and children provided assent. Financial compensation was not provided This study adhered to the Declaration of Helsinki.^34^ The trial protocol and statistical analysis plan are available in Supplement 1.

### Study Sample

Parent-child dyads were enrolled using convenience sampling from March 11, 2019, to January 29, 2020. Hong Kong Chinese children who have survived cancer were eligible for study inclusion if they met the following criteria: (1) aged 9 to 16 years, (2) able to communicate in Cantonese and read Chinese, (3) had completed cancer treatment at least 6 months previously, and (4) had not engaged in regular PA in the previous 6 months. In addition, parents (either father or mother; the primary caregiver) should be able to use a smartphone with an instant messaging application (ie, WhatsApp or WeChat) installed. Children with evidence of recurrence or secondary cancers and those with physical impairment, cognitive impairment, or impaired mental status identified from their medical records were excluded. Parents with emotional or psychiatric disorders and cognitive and learning problems identified from their medical records were also excluded from the study.

### Randomization and Masking

Parent-child dyads were randomized (1:1) to an intervention or control group using a computer-generated list (Research Randomized software, version 14.0; Social Psychology Network) prepared by a research staff member (Dr Ho) not involved in participant recruitment. The allocation sequence was concealed on cards placed inside sequentially numbered, opaque, sealed envelopes, which were opened by a research assistant not involved in recruiting participants or providing the intervention. The outcome assessor was blinded to group allocation to ensure objectivity during the data collection process.

### Sample Size

We used power analysis to estimate the sample size, referring to the results of a pilot study and findings of previous intervention studies conducted by our group on efforts to promote PA among children who have survived cancer.^35,36^ An average moderate effect size (Cohen *d* = 0.5) was found for the outcomes of PA and fatigue levels, handgrip strength (HGS), and QOL. In addition to the consultation with an expert panel (comprising a pediatric oncologist [G.C.-F.C.], a nursing specialist in pediatric oncology, and a professor [W.H.C.L.] and an assistant professor [J.O.K.C.] from local universities), a review of the literature also indicated that this result can be regarded as the minimally clinically important difference that warrants a change in patient management within pediatric populations.^37,38,39,40,41^ To detect a statistically meaningful between-group difference with a moderate effect size (Cohen *d* = 0.5) and a power of 80% (2-tailed; with a potential attrition rate of 20%) at α = .05, a minimum of 160 parent-child dyads with 80 dyads per arm was required.

### Intervention Group

Parent-child dyads in the intervention group received a 10-minute health advice session delivered by a trained research nurse, who had received professional training from a clinical psychologist in delivering brief MI, at the time of recruitment in the pediatric outpatient clinics. The session highlighted the specific health benefits of regular PA for the children. In addition, an individual face-to-face brief MI session of approximately 10 minutes was delivered to parents. This session established a rapport between the nurse and parents to foster communication in the brief MI delivered via instant messaging applications. In addition, the parent-child dyads were invited to visit a website established by the Centre for Health Protection, Department of Health, Hong Kong Special Administrative Region,^42^ which contains information on PA. The nurse then assessed the physical condition of each child to ensure that they were physically fit to perform PA and identify appropriate types of PA for them. Moreover, children were asked about their sports preferences and interests during the assessment to enhance their enjoyment in performing PA.

Individualized brief MI was delivered to each parent via mobile instant messaging applications by the nurse. A menu of strategies was used to deliver brief MI focused on 4 fundamental processes (engaging, focusing, evoking, and planning) to guide parents toward the resolution of ambivalence about their child’s engagement in regular PA) (eTable in Supplement 2). Brief MI was delivered via instant messaging relatively intensively as per parents’ preferences, usually not less than once per week and not more than 3 times per week for the first 6 months. During each communication, parents were asked whether they had encouraged their child to perform regular PA in the past week. After 6 months, minimal messages were delivered to parents to maintain contact until the 12-month follow-up. The messages focused on monitoring their child’s behavior change progress and responding to any questions parents had.

To ensure intervention integrity, the brief MI intervention was delivered by the same nurse throughout the study. Messages delivered to the parents were digitally recorded and periodically reviewed by the research team. Weekly meetings were held to monitor the quality of the intervention implementation.

### Control Group

Control group participants received their usual care at the pediatric oncology clinics. Like the parent-child dyads in the intervention group, those in the control group received the 10-minute health advice session and were invited to browse the website. However, control group parents did not receive a brief MI delivered via instant messaging throughout the study period.

### Study Outcomes

Data collection was performed at baseline (T0) at the outpatient clinics and 1 (T1), 3 (T2), 6 (T3), and 12 (T4) months during home visits after the start of the intervention. The primary outcome was children’s PA levels at 12 months, assessed using the Chinese University of Hong Kong: PA Rating for Children and Youth (CUHK-PARCY), with scores ranging from 0 to 10 (higher scores indicate greater PA levels).^15^ The secondary outcomes comprised cancer-related fatigue levels (measured using the Chinese version of the Fatigue Scale: Child [FS-C], with scores ranging from 13 to 65; higher scores represent greater levels of fatigue^35,36,43^), left and right HGS (measured using a handheld dynamometer, with scores ranging from 0 to 90 kg; higher scores represent greater HGS^44^), PEFR (measured using a mini-Wright Standard Handheld peak flow meter, with scores ranging from 60 to 880 L/min; higher scores represent greater PEFR^45,46,47^), and QOL (measured using the Chinese version of the Pediatric QOL Inventory [PedsQL] 4.0 Generic Core Scales for children, with scores ranging from 0 to 100; higher scores represent better QOL^48^) at 6 and 12 months. The eAppendix in Supplement 2 provides detailed descriptions of each outcome measure. Children completed the CUHK-PARCY, FS-C, and Pediatric QOL Inventory scales and performed the HGS and PEFR tests; parents provided information on the demographic and clinical characteristics of their children.

### Statistical Analysis

Data were analyzed using SPSS statistics software, version 25.0 (IBM Corp), and according to the intention-to-treat principle. Descriptive statistics were used to summarize the demographic characteristics and clinical outcomes of the participants at each time point. The normality of the data was assessed using normal probability plots and skewness statistic. Generalized estimating equation models were used to examine the effect of the intervention on each outcome between groups at T1, T2, T3, and T4 compared with T0 in terms of (1) between-group differences (group effects), (2) within-group changes from baseline (time effects), and (3) between-group differences in change from baseline (group-by-time interaction effects). β refers to the estimated coefficient from the multivariate generalized estimating equations GEE models. The β coefficients of the group-by-time interaction terms represent the difference in mean changes in an outcome at a specific time point with respect to the baseline (T0) between the intervention and control groups (mean change in intervention group − mean change in control group). A positive group-by-time interaction coefficient indicates a greater mean change in intervention group 1 compared with control group. A 5% level of significance was assumed, and all significance tests were 2-sided. Cohen *d* effect sizes were also calculated for the outcomes at each postintervention time point. Markov chain Monte Carlo multiple imputation was used to handle missing data.

## Results

**Figure 2.**
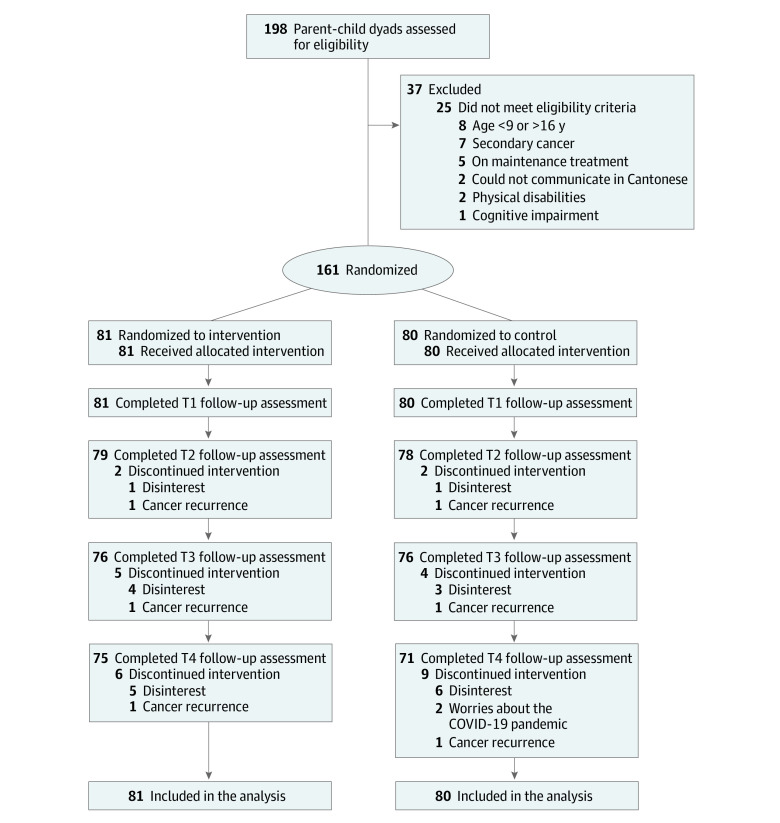
Patient Flow Diagram T1 indicates 1 month after starting the intervention; T2, 3 months after starting the intervention; T3, 6 months after starting the intervention; and T4, 12 months after starting the intervention.

Between March 11, 2019, and January 29, 2021, of 198 parent-child dyads assessed for eligibility, a total of 161 (81.3%) consented to participate and were randomized to the intervention group (n = 81) or the control group (n = 80) (Figure 2). The retention rates at 12 months were 92.6% in the intervention group and 88.6% in the control group.

Table 1 presents the demographic and clinical characteristics of the parent-child dyads. The mean (SD) age of the children was 12.4 (2.4) years (range, 9-16 years), 93 children were boys (57.8%), and 68 were girls (42.2%). Most of the children had been diagnosed with leukemia, lymphoma, or brain tumors (133 [82.6%]). The mean (SD) age of the parents was 42.8 (6.2) years (range, 32-59 years), 115 parents were women (71.4%), and 46 (28.6%) were men. A total of 115 parents (71.4%) had received a high school degree or above. The median number of instant messages delivered to each parent was 365 (range, 310-468).

**Table 1.** Demographic, Clinical, and Baseline Characteristics of the Participants

Characteristic	Group, No. (%)	
	Intervention (n = 81)	Control (n = 80)
Child		
Age, mean (SD), y	12.3 (2.3)	12.4 (2.5)
Sex		
Male	44 (54.3)	49 (61.3)
Female	37 (45.7)	31 (38.8)
Diagnosis		
Leukemia	31 (38.3)	27 (33.8)
Lymphoma	19 (23.5)	21 (26.3)
Tumor		
Brain	20 (24.7)	15 (18.8)
Bone	8 (9.9)	10 (12.5)
Germ-cell and gonadal	3 (3.7)	7 (8.8)
Treatment received		
Surgery	7 (8.6)	10 (12.5)
Chemotherapy	37 (45.7)	36 (45.0)
Radiotherapy	1 (1.2)	2 (2.5)
Bone marrow transplant	5 (6.2)	3 (3.8)
Mixed modality	31 (38.3)	29 (36.3)
Time since treatment was completed, mo		
6-12	31 (38.3)	34 (42.5)
13-24	12 (14.8)	12 (15.0)
25-36	11 (13.6)	9 (11.3)
37-48	9 (11.1)	8 (10.0)
49-60	18 (22.2)	17 (21.3)
Physical activity levels, mean (SD)^a^	2.8 (1.2)	2.8 (1.2)
Cancer-related fatigue, mean (SD)^b^	28.8 (8.8)	28.7 (8.3)
Handgrip strength, mean (SD)^c^		
Left	16.1 (5.8)	16.3 (5.8)
Right	18.3 (6.0)	18.4 (5.8)
Peak expiratory flow rate, mean (SD)^d^	126.1 (40.7)	127.1 (40.1)
Quality of life, mean (SD)^e^	77.0 (12.6)	79.1 (14.2)
Parents		
Age, mean (SD), y	43.8 (5.0)	42.2 (6.1)
Sex		
Male	24 (29.6)	22 (27.5)
Female	57 (70.4)	58 (72.5)
Parents’ educational level		
Primary school or below	3 (3.7)	4 (5.0)
Secondary school		
Lower	20 (24.7)	19 (23.8)
Upper	35 (43.2)	41 (51.3)
Tertiary education	23 (28.4)	16 (20.0)

^a^ Physical activity, measured with the Chinese University of Hong Kong: Physical Activity Rating for Children and Youth. Scores range from 0 to 10 (higher scores indicate greater levels).

^b^ Cancer-related fatigue, measured with the Chinese version of the Fatigue Scale: Child. Scores range from 13 to 65 (higher scores indicate greater levels).

^c^ Handgrip strength, measured with a handheld dynamometer. Scores range from 0 to 90 kg (higher scores indicate greater handgrip strength).

^d^ Peak expiratory flow rate, measured with a mini-Wright Standard Handheld peak flow meter. Scores range from 60 to 880 L/min (higher scores indicate greater peak expiratory flow rate).

^e^ Quality of life, measured with the Pediatric QOL Inventory 4.0 Generic Core Scales. Scores range from 0 to 100 (higher scores represent better quality of life).

### Primary Outcome

Table 2 reports the results of the generalized estimating equation model analysis for the intervention effects on primary outcome variables. For the primary outcome measure (PA levels), a significant main effect was found for the intervention in promoting children’s PA levels (β = 1.95; 95% CI, 1.62-2.23; *P* < .001). Compared with the control group, the intervention group showed significantly greater increments in PA levels at T2, T3, and T4 (group-by-time interaction effect, T2: β = 2.32; 95% CI, 1.92-2.73; *P* < .001; T3: β = 3.09; 95% CI, 2.65-3.53; *P* < .001; and T4: β = 3.91; 95% CI, 3.45-4.36; *P* < .001). Moderate to vigorous PA levels among participants in the intervention group increased by 72.8%, compared with 6.3% in controls, during the 12-month study period. In addition, the mean PA level of the intervention group was approximately 4.2 SD higher than that of the control group at the 12-month follow-up.

**Table 2.** Generalized Estimating Equation Analysis for the Intervention Effects on the Primary Outcomes

Primary outcome	Mean (SD)		Group effect		Time effect		Group × time effect		Cohen *d*
	Intervention (n = 81)	Control (n = 80)	β (95% CI)^a^	*P* value	β (95% CI)^a^	*P* value	β (95% CI)^a^	*P* value	
Physical activity levels^b^									
T0	2.83 (1.24)	2.84 (1.22)	1.95 (1.62 to 2.23)	<.001	NA	NA	NA	NA	0.00
T1	3.11 (1.22)	2.68 (1.12)			−0.16 (−0.34 to 0.01)	.07	−0.15 (−0.51 to 0.21)	.43	0.35
T2	5.21 (1.34)	2.75 (1.16)			1.15 (0.92 to 1.39)	<.001	2.32 (1.92 to 2.73)	<.001	1.99
T3	6.12 (1.33)	2.83 (1.22)			1.53 (1.21 to 1.85)	<.001	3.09 (2.65 to 3.53)	<.001	2.64
T4	6.96 (1.37)	2.69 (1.02)			1.95 (1.56 to 2.33)	<.001	3.91 (3.45 to 4.36)	<.001	3.53

Abbreviations: T0, time of recruitment; T1, 1 month after starting the intervention; T2, 3 months after starting the intervention; T3, 6 months after starting the intervention; T4, 12 months after starting the intervention.

^a^ β refers to the estimated coefficient from the multivariate generalized estimating equations models. The β coefficients of the group-by-time interaction terms represent the difference in mean changes in an outcome at a specific time point with respect to the baseline (T0) between the intervention and control groups (mean change in intervention group − mean change in control group). A positive group-by-time interaction coefficient indicates a greater mean change in intervention group compared with control group.

^b^ Physical activity, measured with the Chinese University of Hong Kong: Physical Activity Rating for Children and Youth. Scores range from 0 to 10 (higher scores indicate greater levels).

### Secondary Outcomes

Table 3 reports the results of the generalized estimating equation model analysis for the intervention effects on secondary outcome variables. Significant main effects were found for the intervention in reducing cancer-related fatigue (β = −3.60; 95% CI, −5.97 to −1.24; *P* = .003), improving left HGS (β = 1.78; 95% CI, 0.05-3.44; *P* = .04), right HGS (β = 2.11; 95% CI, 0.40-3.82; *P* = .02), and QOL (β = 4.00; 95% CI, 0.25-7.74; *P* = .04), whereas no significant improvement was noted for PEFR (β = 6.48; 95% CI, −5.49 to 18.45; *P* = .29).

**Table 3.** Generalized Estimating Equation Analysis for the Intervention Effects on the Secondary Outcomes

Secondary outcomes	Mean (SD)		Group effect		Time effect		Group × time effect		Cohen *d*
	Intervention (n = 81)	Control (n = 80)	β (95% CI)^a^	*P* value	β (95% CI)^a^	*P* value	β (95% CI)^a^	*P* value	
Cancer-related fatigue^b^									
T0	28.78 (8.79)	28.66 (8.26)	−3.60 (−5.97 to −1.24)	.003	NA	NA	NA	NA	NA
T1	28.20 (8.40)	29.06 (8.24)			−0.09 (−0.28 to 0.09)	.32	−0.47 (−3.04 to 2.11)	.72	0.11
T2	26.02 (7.71)	28.82 (8.25)			−1.40 (−1.83 to −0.96)	<.001	−2.71 (−5.16 to −2.52)	<.001	0.34
T3	22.89 (6.85)	28.50 (8.12)			−3.01 (−3.64 to −2.37)	<.001	−5.69 (−8.03 to −3.35)	<.001	0.74
T4	19.49 (5.65)	28.44 (8.39)			−4.84 (−5.80 to −3.89)	<.001	−9.16 (−11.31 to −7.00)	<.001	1.24
Left-hand grip strength^c^									
T0	16.14 (5.79)	16.30 (5.75)	1.78 (0.05 to 3.44)	.04	NA	NA	NA	NA	0.03
T1	16.30 (5.80)	16.29 (5.76)			0.07 (0.04 to 0.10)	<.001	0.004 (−1.78 to 1.79)	>.99	0.00
T2	17.26 (5.75)	16.44 (5.74)			0.58 (0.45 to 0.71)	<.001	1.04 (−0.75 to 2.83)	.25	0.16
T3	19.09 (5.79)	16.47 (5.78)			1.46 (1.02 to 1.91)	<.001	2.69 (0.96 to 4.43)	.002	0.45
T4	22.16 (6.29)	16.72 (5.79)			2.88 (2.16 to 3.60)	<.001	5.52 (3.70 to 7.33)	<.001	0.91
Right-hand grip strength^c^									
T0	18.29 (5.99)	18.40 (5.77)	2.11 (0.40 to 3.82)	.02	NA	NA	NA	NA	0.02
T1	18.52 (5.95)	18.39 (5.76)			0.10 (0.04 to 0.17)	<.001	0.11 (−1.69 to 1.92)	.90	0.02
T2	19.43 (6.00)	18.42 (5.75)			1.59 (1.30 to 1.88)	<.001	2.79 (0.98 to 4.60)	.002	0.17
T3	21.28 (5.75)	18.60 (5.81)			1.49 (1.07 to 1.91)	<.001	2.75 (1.01 to 4.50)	.002	0.47
T4	24.29 (6.16)	18.90 (5.89)			2.87 (2.13 to 3.61)	<.001	5.45 (3.62 to 7.27)	<.001	0.89
Peak expiratory flow rate^d^									
T0	126.11 (40.70)	127.06 (40.05)	6.48 (−5.49 to 18.45)	.29	NA	NA	NA	NA	0.02
T1	126.60 (41.12)	127.38 (39.63)			0.40 (−0.13 to 0.94)	.14	−0.46 (−12.92 to 12.00)	.94	0.02
T2	130.89 (40.64)	127.59 (39.65)			2.64 (1.74 to 3.53)	<.001	3.82 (−8.69 to 16.34)	.55	0.08
T3	139.55 (40.21)	128.94 (39.20)			3.01 (3.95 to 39.78)	<.001	4.33 (−8.05 to 16.71)	.49	0.27
T4	156.91 (41.32)	130.82 (39.52)			15.38 (11.09 to 19.66)	<.001	28.51 (16.10 to 40.92)	<.001	0.65
Quality of life^e^									
T0	77.00 (12.58)	79.14 (14.19)	4.00 (0.25 to 7.74)	.04	NA	NA	NA	NA	0.16
T1	78.60 (12.47)	78.64 (14.08)			0.55 (0.29 to 0.82)	<.001	−0.55 (−4.65 to 3.56)	.79	0.00
T2	79.79 (12.19)	78.53 (13.95)			1.12 (0.75 to 1.63)	<.001	3.81 (−3.26 to 4.87)	.70	0.10
T3	83.73 (10.84)	78.77 (13.63)			3.40 (2.63 to 4.17)	<.001	5.01 (1.19 to 8.82)	.01	0.40
T4	93.30 (6.07)	78.21 (13.77)			7.71 (5.99 to 9.44)	<.001	14.19 (10.84 to 17.54)	<.001	1.42

Abbreviations: T0, time of recruitment; T1, 1 month after starting the intervention; T2, 3 months after starting the intervention; T3, 6 months after starting the intervention; T4, 12 months after starting the intervention.

^a^ β refers to the estimated coefficient from the multivariate generalized estimating equations models. The β coefficients of the group-by-time interaction terms represent the difference in mean changes in an outcome at a specific time point with respect to the baseline (T0) between the intervention and control groups (mean change in intervention group − mean change in control group). A positive group-by-time interaction coefficient indicates a greater mean change in intervention group compared with control group.

^b^ Fatigue, measured with the Chinese version of the Fatigue Scale: Child. Scores range from 13 to 65 (higher scores indicate greater levels).

^c^ Handgrip strength, measured with a handheld dynamometer. Scores range from 0 to 90 kg (higher scores indicate greater handgrip strength).

^d^ Peak expiratory flow rate, measured with a mini-Wright Standard Handheld peak flow meter. Scores range from 60 to 880 L/min (higher scores indicate greater peak expiratory flow rate).

^e^ Quality of life, measured with the Pediatric QOL Inventory 4.0 Generic Core Scales. Scores range from 0 to 100 (higher scores represent better quality of life).

Compared with the control group, the intervention group showed significantly greater improvement in cancer-related fatigue (group-by-time interaction effect, T2: β = −2.71; 95% CI, −5.16 to −2.52; *P* < .001; T3: β = −5.69; 95% CI, −8.03 to −3.35; *P* < .001; T4: β = −9.16; 95% CI, −11.31 to −7.00; *P* < .001), left HGS (T3: β = 2.69; 95% CI, 0.96-4.43; *P* = .002; T4: β = 5.52; 95% CI, 3.70-7.33; *P* < .001), right HGS (T2: β = 2.79; 95% CI, −0.98 to 4.60; *P* = .002; T3: β = 2.75; 95% CI, 1.01-4.50; *P* = .002; T4: β = 5.45; 95% CI, 3.62-7.27; *P* < .001), PEFR (T4: β = 28.51; 95% CI, 16.10-40.924; *P* < .001), and QOL (T3: β = 5.01; 95% CI, 1.19-8.82; *P* = .01; T4: β = 14.19; 95% CI, 10.84-17.54; *P* < .001).

## Discussion

This randomized clinical trial showed that delivering brief MI to parents of children who survived cancer via mobile instant messaging applications substantially increased their children’s PA levels. In addition, the difference in the mean PA level of the intervention group (approximately 4.2 SD higher than that of the control group) was greater than the recommendations for minimally clinically important difference in patient-reported outcomes (0.25-0.33 SD) and exceeds the clinically meaningful indicator of intervention efficacy.^37,38,39,40,41,49^ Intervention effects remained up to 12 months after the 6-month brief MI intervention. This study explored a sustainable means to address the problem of physical inactivity in children who survive cancer, which is a leading public health issue worldwide.

Evidence supports the efficacy of parent-based interventions for improving children’s health-enhancing behaviors, including PA.^1,50,51^ This study extended previous research and, to our knowledge, was the first randomized clinical trial to use brief MI to motivate parents to encourage their children surviving cancer to engage in regular PA. The intervention may have been effective because it alleviated parents’ concerns and misconceptions about their child’s engagement in PA, thus substantially enhancing parents’ motivation to promote their child’s engagement in regular PA. Moreover, family involvement is important in Chinese culture with an emphasis on children following parental instructions and advice.^23^ Some previous PA interventions have not engaged families in promoting PA among the pediatric oncology population.^35,36^ Our study used mobile instant messaging applications to deliver brief MI to facilitate parental engagement. The use of this technology enables direct, real-time, continuing professional counseling and support for parents; the rapid delivery of instant messaging provided a means of 2-way communication that was flexible, efficient, and time-saving.^33,52^ By portraying a positive attitude toward PA, the intervention increased parents’ willingness to act as role models and play a supporting role by facilitating PA for their child.

Cancer-related fatigue is the most distressing and persistent symptom of pediatric cancer.^6^ A baseline mean fatigue score of 28.7 was reported by the PCS in this study, which was higher than that of healthy Hong Kong Chinese children of similar ages, who have a mean fatigue score of 22.6.^43^ Approximately 73% of the participants in our study had a fatigue score of 22.6 or above, indicating that many children experienced higher levels of cancer-related fatigue than their healthy counterparts.^43^ The significant improvement in cancer-related fatigue in intervention group participants at the 3-, 6-, and 12-month follow-ups can be mostly attributed to the increase in PA levels throughout the intervention period. Our findings also showed that the mean level of cancer-related fatigue among intervention group participants at the 12-month follow-up was similar to that of their healthy counterparts.^43^ These findings provide additional evidence of the clinical significance of the brief MI in promoting PA, which could help to attenuate cancer-related fatigue.

Despite the present study not showing a significant between-group effect on PEFR, the PEFR of the intervention group participants increased gradually across the 5 time periods, with a significant difference at the 12-month follow-up. This improvement suggests that, although the intervention does not have an immediate effect on PEFR, a positive effect may become apparent after a longer period. Future studies should extend the follow-up period to determine the long-term effect of the intervention on the pulmonary function of children who survive cancer.

### Strengths and Limitations

The main study strength was its design; this was an assessor-masked, multicenter randomized clinical trial with a large sample and adequate statistical power to detect a clinically meaningful effect. Other strengths include low attrition, a strong theoretical framework to guide the intervention, and multiple follow-up times.

This study had limitations. Selection bias may have occurred because convenience sampling was used. Follow-up was conducted only up to 12 months; therefore, the long-term effects of the brief MI intervention on promoting regular PA in the children remain unclear. Further investigation of the long-term sustainability of the outcomes of such an intervention is warranted. A validated and reliable CUHK-PARCY scale was adopted to evaluate the children’s PA levels, yet potential reporting bias may exist. Future research might consider using objective measures, such as pedometers or accelerometers, to assess the participants’ PA levels. This study relied solely on child self-reports to assess the outcome measures owing to time constraints during the medical follow-ups. Parent proxy reports on the outcome measures may be included in future research to provide a comparison with child self-report measures. Children with cognitive and physical impairments were excluded from the study, which may limit the generalizability of the findings to the whole pediatric cancer survivor population.

## Conclusions

In this randomized clinical trial, a brief MI intervention delivered to parents via mobile instant messaging applications was effective in promoting the adoption and maintenance of regular PA among Chinese children who survived cancer. The intervention can be integrated into pediatric survivorship care to attenuate cancer- and treatment-related adverse effects and improve QOL among the vulnerable pediatric oncology population.

## Notes

Supplement 1.
Trial Protocol and Statistical Analysis Plan

Supplement 2. **eTable.** The Menu of Strategies of Brief Motivational Interviewing **eAppendix.** A Detailed Description of Each Measure Used in This Trial
eReferences

Supplement 3.
Data Sharing Statement
