# Supplementary material for: Efficacy of Mobile Instant Messaging–Delivered Brief Motivational Interviewing for Parents to Promote Physical Activity in Pediatric Cancer Survivors: A Randomized Clinical Trial
Source: JAMA Netw Open. 2022 Jun 14;5(6):e2214600. doi: 10.1001/jamanetworkopen.2022.14600 (PMC9198728; doi:10.1001/jamanetworkopen.2022.14600)
Supplement: Supplement 1. — Trial Protocol and Statistical Analysis Plan [file jamanetwopen-e2214600-s001.pdf]

Supplement 1. Original trial protocol and statistical analysis plan

Title

Efficacy of Mobile Instant Messaging-Delivered Brief Motivational Interviewing for Parents to Promote Physical Activity in Pediatric Cancer Survivors: A Randomized Clinical Trial

Background and Introduction

With advancements in diagnostic techniques, cancer treatments and adjunctive therapies, the overall survival rate of pediatric brain tumors have been remarkably increased. However, numerous studies have indicated that the improved pediatric cancer survival has inevitably been accompanied by increased chronic physical problems and adverse psychological late-effects of cancer and its treatment.[1-3] The frequently reported physical and psychological sequelae included cancer-related fatigue, reduced muscle strength, decreases in functional capacity, activity intolerance, depression and distorted self-esteem, which in turn severely comprises their quality of life (QoL). [1, 4-6] Among these sequelae, cancer-related fatigue is the most common problem experienced by pediatric cancer survivors.[7-9] Substantial evidence has revealed that adaptation in regular physical activity can reduce cancer-related fatigue, enhance muscle strength and improve functional capacity.[10-11] Nevertheless, considerable concern has been raised towards the declining levels of physical activity among pediatric cancer survivors.[12] In a local cross-sectional study which assessed the physical activity and the factors affecting regular exercise among 128 young Hong Kong Chinese cancer survivors revealed that their current physical activity levels were markedly reduced when compared with the pre-morbid situation. The study also showed that cancer-related fatigue is the most significant factor that prevented the survivors from engaging in regular physical activity.[12] Alarmingly, evidence shows that physical inactivity can lead to a wide array of health problems, including muscle catabolism and atrophy and

cardiovascular diseases, which in turn may further exaggerate cancer-related fatigue and lead to a reduction in pediatric cancer survivors' functional capacity, hence causing long-term decrement in their QoL.[12] In light of the breadth and long lasting nature of those aforementioned negative impacts of cancer, healthcare professionals should therefore take a proactive role in offering prompt support and developing appropriate interventions that can help reduce fatigue among young cancer survivors, and increase their awareness of the importance of regular physical activity.

A large body of evidence supports that regular physical activity has beneficial effects on both physical and psychological well-being of pediatric cancer survivors.[13-15] In addition, evidence shows that engaging in regular moderate-intensity physical activity may help ameliorate some of the treatment-related adverse effects experienced by pediatric cancer survivors. Such benefits include reduced cancer-related fatigue and improved physical fitness, functional capacity, muscle strength, immunity, and bone health, all of which contribute to an overall reduced risk of morbidity and an enhancement in QoL.[14] Given the long-term health benefits associated with regular, moderate levels of physical activity, there is an imperative need to advocate the idea of having regular physical activity in pediatric cancer survivors. To this end, the development and evaluation of appropriate strategies that can increase the adoption and maintaining of regular physical activity among pediatric cancer survivors is crucial.

There are several reasons to dissect why Hong Kong Chinese pediatric cancer survivors reported substantially low physical activity levels. First, due to the influence of the philosophy of Confucianism, which focuses on the maintenance of harmony in our bodies to attain health, survivors' parents or healthcare professionals may held the misconception that physical activity. As children exhibited fatigue after their recovery from cancer, which may have led parents to

be hesitant to encourage their children to perform energy-consuming activities such as physical activity.[12,16] Thus, pediatric cancer survivors are often advised to take more rest to avoid cancer- or treatment-related fatigue. Further, many pediatric cancer survivors show reluctance to perform PA, and Chinese parents may be gentler and more tolerant to this attitude after their child's recovery. Nevertheless, physical inactivity can accelerate fatigue as it induces muscle catabolism and atrophy, which in turn may lead to a further decrease in functional capacity.[17,18] Second, owing to the relatively low body resistance of pediatric cancer survivors, they are often discouraged by their parents to go to crowded public areas, and hence this may hinder their engagement in physical activity. Third, in the society of Hong Kong, rooted in the Confucian traditions, most of the Chinese parents perceive achievement in academic and other domains, such as music and art, as important indicators for determining their child's capability and future possibilities.[19,20] In addition, due to the extremely competitive environment for education in Hong Kong, parents always strive for opportunities to gain educational advantages for their child to maximize their child's probability of enrolling in an elite school.[21] Therefore, Hong Kong Chinese pediatric cancer survivors are required to make extra efforts to catch up with their studies after the suspension from school during the treatment. Consequently, there will be less time available for pediatric cancer survivors to engage in regular physical activity. Further, most Chinese pediatric cancer survivors reported that there is lack of encouragement by their important ones and role models to follow and learn the appropriate physical activities for them to engage in. Indeed, parents are influential in affecting the activities that the children performed,[22] and parental psychological control may inhibit the development of positive mental well-being and affect emotional life in Chinese children. Therefore, it is crucial to understand the perception of engagement of regular physical activity of the pediatric cancer survivors from their parents'

perspectives, with the aim at developing appropriate intervention for the pediatric cancer survivors to adopt and maintain regular physical activity.

The decline in physical activity levels among Hong Kong Chinese pediatric cancer survivors may be largely due to the overlook of the significance of regular physical activity by survivors' parents and their misconception about performing physical activity during their child's survivorship, which all resulted in exhibiting ambivalence and low motivation to encourage their children engaging in regular physical activity.[12] Thus, it is imperative for the healthcare professionals to instill proper concept to the parents regarding the adoption and maintenance of regular physical activity, with the aim of enhancing Hong Kong Chinese pediatric cancer survivors' physical well-being and improving their QoL.

A substantial body of evidence has demonstrated that education alone is insufficient or unlikely to change behavior.[23] MI is a directive, client-centered counseling strategy which differs from prevailing patient education to support clients to explore and resolve their ambivalence about changing their behaviors.[24] MI has demonstrated efficacy and effectiveness in promoting positive health behavioral changes, for instance, oral health,[25,26] alcohol and other substance use.[27-29] Yet, traditional MI requires an intensive treatment, which may not be feasible to be implemented in the Hong Kong context due to the busy life. A brief MI that emphasises the adoption of shorter and simpler style of counselling was thus developed.[30] MI involves elicitation of individual's motivation, enhancement of individual's commitment, and exploration of solutions toward change of behavior through four stages, namely engaging, focusing, evoking and planning.[31] Engaging involves the process of establishing a collaborative relationship with the client by expressing empathy through reflective listening.[31] Focusing is the ongoing process of guiding the client to identify a specific area about which the client is ambivalent to make a

change.[31] Evoking focuses the elicitation of the client's intrinsic motivation for change by developing discrepancy between the client's goals or values and his or her current behavior.[32] Planning involves making and consolidation of a commitment to change as well as developing an action plan to adapt to a new behavior.[31]

Rationales of using mobile instant messaging applications as a mean to deliver brief motivational interviewing

There is an increase in the use of information communication technology (ICT) for health care delivery and health promotion.[31-34] According to the definition by the World Health Organization, mobile health refers to medical and public health practice which are supported by mobile devices using instant messaging (IM) applications, such as WhatsApp or WeChat.[35] ICT are expanding rapidly worldwide, it is a new strategy to promote health.[36] Using IM applications (i.e. WhatsApp or WeChat) allows quick, direct and continuing professional advice and support for the parents to manage their child's level of physical activity. Instant messaging can be delivered instantaneously that can be accessed at a time that suits recipients. In addition, instant messaging is more cost-effective than face-to-face meetings and offers for mutual communication, in which parents can elicit feedback and interact flexibly.[36]

Research Plan and Methodology

Study design

An assessor-blinded prospective RCT will be conducted in two pediatric outpatient clinics in two public hospitals in different districts of Hong Kong. The aim of the RCT is to examine the effects of a brief MI delivered to parents via mobile instant messaging on PA levels, cancer-related fatigue, handgrip strength (HGS), peak expiratory flow rates (PEFR), and QoL among Chinese pediatric cancer survivors. Participants will be recruited from the two pediatric

oncology outpatient clinics in Hong Kong. The parent-child dyads who meet the criteria will undergo baseline assessments before randomization. The method of simple complete randomization will be adopted. Subjects will be randomly allocated to experimental or control groups. Randomization will be performed by a research assistant opening a sequentially numbered, opaque sealed envelope with a card inside indicating the allocated group. The random numbers to be used for group assignment will be computer-generated by another research assistant before subjects recruitment begins. Allocation concealment will thus be ensured.

Subjects

a) Pediatric cancer survivors

Hong Kong pediatric cancer survivors meeting the inclusion criteria will be invited to participate in this study. The inclusion criteria included (1) children aged from 9 to 16 years, (2) able to communicate in Cantonese and read Chinese, (3) have completed treatment for at least six months previously, (4) does not participate in physical exercise in the previous 6 months. Survivors with evidence of recurrence or secondary malignancies and those with physical impairment, cognitive impairment, or impaired mental status identified from their medical records will be excluded.

b) Parents of pediatric cancer survivors (either father or mother; the primary caregiver of pediatric cancer survivors)

Parents of pediatric cancer survivors must be (1) able to speak Cantonese and read Chinese, (2) able to use a smartphone with an IM application (i.e., WhatsApp or WeChat) installed. Parents with emotional or psychiatric disorders, and cognitive and learning problems identified from their medical records will be excluded from the study.

Sample size calculation

We used power analysis to estimate the sample size, referring to the results of our previous pilot study of the intervention and findings of our previous intervention studies on efforts to promote PA among pediatric cancer survivors in Hong Kong.[37,38] An average moderate effect size (Cohen's *d* = 0.5) was found for the outcomes of PA and fatigue levels, muscle strength, and QoL. Consultation with an expert panel, comprising a pediatric oncologist, a nursing specialist in pediatric oncology, and a professor and assistant professor from a local university, indicated that this result can be regarded as the minimally important difference that warrants a change in patient management. To detect a statistically meaningful between-group difference with a moderate effect size (Cohen's *d* = 0.5) and a power of 80% (two-tailed; with a potential attrition rate of 20%) at α=.05, a minimum of 160 parent–child dyads with 80 dyads per arm will be required.

Intervention

(a) Intervention group

As well as receiving scheduled medical follow-ups at the oncology outpatient clinic, children and parents will receive BMI and instant messaging delivered by a trained research nurse (RA).

In pediatric outpatient clinic

At the time of recruitment, both children and parents will receive a 10-minute health advice session on the significance of and misconceptions about regular physical activity for cancer survivors and strategies for overcoming barriers to engaging in physical activity delivered by a trained research nurse at the time of recruitment in the pediatric outpatient clinics. Parents will then receive a face-to- face brief MI session of approximately 10 minutes. In this session, parents will also be encouraged to motivate their children to intensify their physical activity levels progressively, with the ultimate goal at achieving the Global Recommendations on Physical

Activity on Health suggested by the World Health Organization. Additionally, they will be invited to visit a website from the Centre for Health Protection, Department of Health, HKSAR website (https://www.chp.gov.hk/en/healthtopics/content/25/8804.html) that contains information on physical activity. A comprehensive assessment will be conducted to children to identify appropriate types of physical activity for them by a research nurse who was trained by one of the coinvestigators, who is an assistant professor specializing in sports and recreation management. The assessment may involve assessing children's physical condition, for instance functional mobility, walking gait and balance. Children's preference on types of physical activity will also be discussed. All parents will be informed that they will receive an individual BMI intervention and instant messaging to facilitate the engagement in physical activity of their children via WhatsApp/WeChat on the smartphones throughout the study period.

Follow-up booster intervention

Personalized brief MI will be delivered to each parent via WhatsApp/WeChat on a smartphone by the research nurse, who have received professional training from a clinical psychologist in delivering brief MI. The brief MI will be delivered based upon the use of a menu of strategies. The brief MI will be delivered more intensively as preferred by the parents, usually not less than once every week and not more than three times per week for the first 6 months. The frequency of delivering messages via WhatsApp/WeChat will be interactive, depending on the parents' actions and responses, their time availability as well as their progress. However, the total time spent by the interventionist would not be more than that for a traditional MI with several long sessions. After 6 months, minimal messages will be provided to the parents by merely following their progress of behavioral changes and responding to their questions to maintain contact until the 12-month follow-up.

Ensuring intervention integrity

To ensure intervention integrity, all brief MI will be conducted by the same registered nurse via mobile instant messaging applications. The research team will periodically check the recruitment and delivery of the intervention by reviewing the messages in WhatsApp/WeChat. In addition, a weekly research team meeting will be held to evaluate the quality of intervention implementation. All brief MI sessions will be digitally recorded for review, but the subjects' personal information will remain confidential and anonymous.

(b) Control group

As well as receiving usual care at the pediatric oncology outpatient clinics, like the parent-child dyads in the experimental group, those in the control group will receive the 10-min health advice session on physical activity and ask to visit the website that contains information on physical activity at the time of recruitment similar to the intervention group. However, parents will not receive brief MI throughout the study period.

Outcome measures

The primary outcome measure is the level of physical activity of the survivors at 12 months after starting the intervention. The secondary outcome measures are survivors' levels of fatigue, left- and right-HGS, PEFR and QoL at baseline, 1, 3, 6, and 12 months after starting the intervention. Participants in both groups will be required to complete the questionnaires and assessments at baseline (during their medical follow-up), 1, 3, 6 and 12 months after starting the intervention (during home visits).

Measuring outcomes

(1) Physical activity level

The Chinese University of Hong Kong: Physical Activity Rating for Children and Youth (CUHK-PARCY)

The subjects' levels of physical activity will be assessed by the CUHK-PARCY. It comprises only one item adapted from two validated scales – the Jackson Activity Coding[39] and the Godin-Shephard Activity Questionnaire Modified for Adolescents.[40] The subjects will be asked to rate their overall weekly levels of physical activity over the past six months from 0 (no exercise at all) to 10 (vigorous exercise on most days), taking into account the frequency, duration and intensity of the activity concerned. Scores of 0-2, 3-6 and 7-10 represent low, moderate and high physical activity levels, respectively.

(2) Level of fatigue

The Chinese version of the Fatigue Scale – Child (FS-C)

The Chinese version of the FS-C will be used to assess the level of fatigue of subjects aged from 7 to 12.[41] It consists of 14 items, evaluated on a five-point Likert Scale, with scores ranging from 14 to 70. Higher scores represent higher levels of cancer-related fatigue.

(2) Left- and right-handgrip strength

Hand-held dynamometer (HHD) will be used to assess the survivors' right- and left-HGS. Before the measurement, the handle of the HHD will be adjusted to allow the base to rest on the first metacarpal (heel of palm) and the handle to rest on the middle of four fingers. During the test, subjects will be instructed to push as hard as they could, with their arms keeping at right angles and their elbows by the side of their bodies. The subjects will be asked to repeat the test three times with 10 to 20 seconds resting time in between each push. Only the highest of the three measurements in each subjects will be recorded.[42]

(4) Peak expiratory flow rates

A mini-Wright Standard Handheld peak flow meter will be used to determine survivors' PEFR.[43-35] It is a useful physiological test to assess an individual's lung functionality by measuring an individual's maximum speed of expiration. It was shown to be an accurate and precise device to determine an individual's pulmonary functionality.

(6) Quality of life

Survivors' QoL will be assessed using the Chinese version of the Pediatric Quality of Life Inventory (PedsQL).[46] This scale has 23 items rated on a 5-point Likert scale. These items are categorized into four domains: physical functioning (eight items), emotional functioning (five items), social functioning (five items) and school functioning (five items). Each item's score was then reverse scored and linearly transformed into a scale of 0 to 100 (0 = 100, 1 = 75, 2 = 50, 3 = 25, 4 = 0). The range of possible scores is 0–100, with higher scores representing better QoL.

Data collection

To minimize attrition bias, intention-to-treat analysis will be used. Ethical approval will be obtained from the Institutional Review Board of the University of Hong Kong/Hospital Authority Hong Kong West Cluster and Hong Kong Children's Hospital Research Ethics Committee. Before commencement, the Chief of Service of the pediatric oncology unit will be briefed on the purpose, nature and protocol of this study. To identify potential subjects, the research assistant will approach pediatric cancer survivors who have their medical follow-ups in the pediatric outpatient clinic and ask them to indicate their willingness to participate in this study. After checking for eligibility, the research student will explain the study details, such as its nature, purpose, implications, and data collection procedure to each pediatric cancer survivors and their parents. If both the parent and the child agreed to participate, written consent will be obtained from the parents and the child's assent will be also obtained. The children and their

parents will be assured that their participation is completely voluntary and that all of the information provided will be kept confidential. The issue of confidentiality is the major ethical issue, and will be solved by recording the data in a manner that does not allow the participants to be identified (ie. using a non-recognizable code for each patient). This study will be conducted in compliance with the principle of Declaration of Helsinki and ICH-GCP.

Data Handling and Record Keeping

To protect patient privacy, all research data would be handled in line with HA/Hospital's policy in handling/storage/destruction of patients' medical records. They would be locked in cabinets where the department or ward keeps patients' confidential information. Electronic data should be saved in secured computer of the hospital with restricted access. Files containing the audio records will be password-protected and encrypted and closed when computers will be left unattended. All electronic data will be stored in password-protected computers or files.

Data analysis

The Statistical Package for Social Sciences (SPSS) software, version 25.0 for Windows (IBM Corp., Armonk NY, USA) will be used to analyze the data; all significance tests will be performed at a significance level of .05 (two-sided). Descriptive statistics will be used to calculate the mean scores, standard deviations, and score ranges of the various scales. The comparability of the two groups will be assessed using inferential statistics, including independent-samples *t*-tests and chi-squared tests. The normality of the data will be assessed using a normal probability plot and skewness statistic. Generalized estimating equation models will be used to examine the effect of the intervention on each outcome between groups over time; between-group effects, within-group (time) effects, and interaction effects (group-by-time) will be examined.

Implications

It is anticipated that using ICT to deliver brief MI with parents can promote the adoption and maintenance of regular physical activity, thereby improving the physiological outcomes as well as QoL among Hong Kong Chinese pediatric cancer survivors. Most importantly, this study will raise the awareness of the significance of engaging in regular physical activity in promoting physical well-being for pediatric cancer survivors and their parents.

References

1. Dietz AC, Mulrooney DA. Life beyond the disease: relationships, parenting, and quality of life among survivors of childhood cancer. *Haematologica*. 2011;96(5):643-645. doi:10.3324/haematol.2011.042606
2. Li HC, Williams PD, Lopez V, Chung JO, Chiu SY. Relationships among therapy-related symptoms, depressive symptoms, and quality of life in Chinese children hospitalized with cancer: an exploratory study. *Cancer Nurs*. 2013;36(5):346-354. doi:10.1097/NCC.0b013e31824062ec
3. Zeltzer LK, Lu Q, Leisenring W, et al. Psychosocial outcomes and health-related quality of life in adult childhood cancer survivors: a report from the childhood cancer survivor study. *Cancer Epidemiol Biomarkers Prev*. 2008;17(2):435-446. doi:10.1158/1055-9965.EPI-07-2541
4. Gawade PL, Hudson MM, Kaste SC, et al. A systematic review of selected musculoskeletal late effects in survivors of childhood cancer. *Curr Pediatr Rev*. 2014;10(4):249-262. doi:10.2174/1573400510666141114223827

5. Li HC, Chung OK, Ho KY, Chiu SY, Lopez V. A descriptive study of the psychosocial well-being and quality of life of childhood cancer survivors in Hong Kong. *Cancer Nurs*. 2012;35(6):447-455. doi:10.1097/NCC.0b013e31823fcb53
6. Zeltzer LK, Recklitis C, Buchbinder D, et al. Psychological status in childhood cancer survivors: a report from the Childhood Cancer Survivor Study. *J Clin Oncol*. 2009;27(14):2396-2404. doi:10.1200/JCO.2008.21.1433
7. Kestler SA, LoBiondo-Wood G. Review of symptom experiences in children and adolescents with cancer. *Cancer Nurs*. 2012;35(2):E31-E49. doi:10.1097/NCC.0b013e3182207a2a
8. Li HC, Lopez V, Joyce Chung OK, Ho KY, Chiu SY. The impact of cancer on the physical, psychological and social well-being of childhood cancer survivors. *Eur J Oncol Nurs*. 2013;17(2):214-219. doi:10.1016/j.ejon.2012.07.010
9. Yeh CH, Man Wai JP, Lin US, Chiang YC. A pilot study to examine the feasibility and effects of a home-based aerobic program on reducing fatigue in children with acute lymphoblastic leukemia. *Cancer Nurs*. 2011;34(1):3-12. doi:10.1097/NCC.0b013e3181e4553c
10. Lucía A, Earnest C, Pérez M. Cancer-related fatigue: can exercise physiology assist oncologists?. *Lancet Oncol*. 2003;4(10):616-625. doi:10.1016/s1470-2045(03)01221-x
11. Whitsett SF, Gudmundsdottir M, Davies B, McCarthy P, Friedman D. Chemotherapy-related fatigue in childhood cancer: correlates, consequences, and coping strategies. *J Pediatr Oncol Nurs*. 2008;25(2):86-96. doi:10.1177/1043454208315546
12. Chung OK, Li HC, Chiu SY, Ho KY, Lopez V. The impact of cancer and its treatment on physical activity levels and behavior in Hong Kong Chinese childhood cancer survivors. *Cancer Nurs*. 2014;37(3):E43-E51. doi:10.1097/NCC.0b013e3182980255

13. Penedo FJ, Dahn JR. Exercise and well-being: a review of mental and physical health benefits associated with physical activity. *Curr Opin Psychiatry*. 2005;18(2):189-193. doi:10.1097/00001504-200503000-00013

14. Paxton RJ, Jones LW, Rosoff PM, Bonner M, Ater JL, Demark-Wahnefried W. Associations between leisure-time physical activity and health-related quality of life among adolescent and adult survivors of childhood cancers. *Psychooncology*. 2010;19(9):997-1003. doi:10.1002/pon.1654

15. Huang TT, Ness KK. Exercise interventions in children with cancer: a review. *Int J Pediatr*. 2011;2011:461512. doi:10.1155/2011/461512

16. Li WHC. The importance of incorporating cultural issues into nursing interventions for Chinese populations. In: Chien WT, ed. *Strategies in evaluation of complex health care interventions for people with physical or mental health issues*. New York, NY: Nova Biomedical Book; 2009:127-137.

17. Braam KI, van Dijk EM, Veening MA, et al. Design of the Quality of Life in Motion (QLIM) study: a randomized controlled trial to evaluate the effectiveness and cost-effectiveness of a combined physical exercise and psychosocial training program to improve physical fitness in children with cancer. *BMC Cancer*. 2010;10:624. Published 2010 Nov 11. doi:10.1186/1471-2407-10-624

18. Lucía A, Earnest C, Pérez M. Cancer-related fatigue: can exercise physiology assist oncologists?. *Lancet Oncol*. 2003;4(10):616-625. doi:10.1016/s1470-2045(03)01221-x

19. Chen JJ, Chen T, Zhang XX. Parenting styles and practices among Chinese immigrant mothers with young children. *Early Child Dev Care*. 2012; 182:1–21. doi: 10.1080/03004430.2010.533371

20. Huntsinger CS, Huntsinger PR, Ching WD, Lee CB. Understanding cultural contexts fosters sensitive care giving of Chinese American children. *Young Child*. 2000; 55:7–12.

21. Wong YL. How middle-class parents help their children obtain an advantaged qualification: A study of strategies of teachers and managers for their children's education in Hong Kong before the 1997 handover. *Sociol Res Online*. 2007; 12(6). doi.org/10.5153/sro.1638

22. rost S, Loprinzi P. Parental Influences on Physical Activity Behavior in Children and Adolescents: A Brief Review. *Am J Lifestyle Med*. 2011; 5(2):171-181. doi.org/10.1177/1559827610387236

23. Rosen L, Zucker D, Brody D, Engelhard D, Manor O. The effect of a handwashing intervention on preschool educator beliefs, attitudes, knowledge and self-efficacy. *Health Educ Res*. 2009;24(4):686-698. doi:10.1093/her/cyp004

24. Rollnick S., Miller WR. What is motivational interviewing? Behav Cogn Psychother. 1995; 23:315–314.'

25. Cook PF, Richardson G, Wilson A. Motivational interviewing training to promote Head Start children's adherence to oral health care recommendations: results of a program evaluation. *J Public Health Dent*. 2013;73(2):147-150. doi:10.1111/j.1752-7325.2012.00357.x

26. Wu L, Gao X, Lo ECM, Ho SMY, McGrath C, Wong MCM. Motivational Interviewing to Promote Oral Health in Adolescents. *J Adolesc Health*. 2017;61(3):378-384. doi:10.1016/j.jadohealth.2017.03.010

27. Hettema J, Steele J, Miller WR. Motivational interviewing. Ann Rev Clin Psychol. 2005; 1:91–111.

28. Lundahl B, Burke BL. The effectiveness and applicability of motivational interviewing: a practice-friendly review of four meta-analyses. *J Clin Psychol*. 2009;65(11):1232-1245. doi:10.1002/jclp.20638

29. Lundahl B, Moleni T, Burke BL, et al. Motivational interviewing in medical care settings: a systematic review and meta-analysis of randomized controlled trials. *Patient Educ Couns*. 2013;93(2):157-168. doi:10.1016/j.pec.2013.07.012

30. Rollnick S, Heather N, Bell A. Negotiating behaviour change in medical settings: the development of brief motivational interviewing. *J Ment Health*. 1992; 1:25-37. doi.org/10.3109/09638239209034509

31. Miller WR, Rollnick S. *Motivational Interviewing: preparing people to change addictive behavior.* New York, US: Guilford Press; 1991

32. Free C, Knight R, Robertson S, et al. Smoking cessation support delivered via mobile phone text messaging (txt2stop): a single-blind, randomised trial. *Lancet*. 2011;378(9785):49-55. doi:10.1016/S0140-6736(11)60701-0

33. Free C, Phillips G, Galli L, et al. The effectiveness of mobile-health technology-based health behaviour change or disease management interventions for health care consumers: a systematic review. *PLoS Med*. 2013;10(1):e1001362. doi:10.1371/journal.pmed.1001362

34. Lester RT, Ritvo P, Mills EJ, et al. Effects of a mobile phone short message service on antiretroviral treatment adherence in Kenya (WelTel Kenya1): a randomised trial. *Lancet*. 2010;376(9755):1838-1845. doi:10.1016/S0140-6736(10)61997-6

35. mHealth: New horizons for health through mobile technologies: second global survey on eHealth. WHO Global Observatory for eHealth. https://apps.who.int/iris/handle/10665/44607. Updated 2011. Accessed June 22, 2019.

36. Kaplan WA. Can the ubiquitous power of mobile phones be used to improve health outcomes in developing countries?. *Global Health*. 2006;2:9. Published 2006 May 23. doi:10.1186/1744-8603-2-9

37. Li HC, Chung OK, Ho KY, Chiu SY, Lopez V. Effectiveness of an integrated adventure-based training and health education program in promoting regular physical activity among childhood cancer survivors. *Psychooncology*. 2013;22(11):2601-2610. doi:10.1002/pon.3326

38. Li WHC, Ho KY, Lam KKW, et al. Adventure-based training to promote physical activity and reduce fatigue among childhood cancer survivors: A randomized controlled trial. *Int J Nurs Stud*. 2018;83:65-74. doi:10.1016/j.ijnurstu.2018.04.007

39. Baumgartner TA, Jackson AS, eds. Measurement for Evaluation in Physical Education and Exercise Science, 6th ed. Boston, MA: WCB McGraw-Hill; 1996.

40. Godin G, Shephard RJ. A simple method to assess exercise behavior in the community. *Can J Appl Sport Sci*. 1985;10(3):141-146.

41. Ho KY, Li WH, Lam KW, Chiu SY, Chan CF. The Psychometric Properties of the Chinese Version of the Fatigue Scale for Children. *Cancer Nurs*. 2016;39(5):341-348. doi:10.1097/NCC.0000000000000297

42. Hand Grip Strength Protocol. The Center for Drug Abuse and AIDS Research; https://cdaar.tufts.edu/protocols/Handgrip.pdf. Updated 2003. Accessed August 12, 2018.

43. Gupta S, Mittal S, Kumar A, Singh KD. Peak expiratory flow rate of healthy school children living at high altitude. *N Am J Med Sci*. 2013;5(7):422-426. doi:10.4103/1947-2714.115781

44. Jones KP, Mullee MA. Measuring peak expiratory flow in general practice: comparison of mini Wright peak flow meter and turbine spirometer. *BMJ*. 1990;300(6740):1629-1631. doi:10.1136/bmj.300.6740.1629

45. Koyama H, Nishimura K, Ikeda A, Tsukino M, Izumi T. Comparison of four types of portable peak flow meters (Mini-Wright, Assess, Pulmo-graph and Wright Pocket meters). *Respir Med*. 1998;92(3):505-511. doi:10.1016/s0954-6111(98)90299-2

46. Chan LF, Chow SM, Lo SK. Preliminary validation of the Chinese version of the Pediatric Quality of Life Inventory. *Int J Rehabil Res*. 2005;28(3):219-227. doi:10.1097/00004356-200509000-00004
