# Supplementary material for: Efficacy of Mobile Instant Messaging–Delivered Brief Motivational Interviewing for Parents to Promote Physical Activity in Pediatric Cancer Survivors: A Randomized Clinical Trial
Source: JAMA Netw Open. 2022 Jun 14;5(6):e2214600. doi: 10.1001/jamanetworkopen.2022.14600 (PMC9198728; doi:10.1001/jamanetworkopen.2022.14600)
Supplement: Supplement 2. — eTable. The Menu of Strategies of Brief Motivational Interviewing eAppendix. A Detailed Description of Each Measure Used in This Trial eReferences [file jamanetwopen-e2214600-s002.pdf]

## Supplementary Online Content

Cheung AT, Li WHC, Ho LLK, Chan GCF. Lam HS, Chung JOK. Efficacy of mobile instant messaging–delivered brief motivational interviewing for parents to promote physical activity in pediatric cancer survivors: a randomized clinical trial. *JAMA Netw Open*. 2022;5(6):e2214600. doi:10.1001/jamanetworkopen.2022.14600

**eTable.** The Menu of Strategies of Brief Motivational Interviewing

**eAppendix.** A Detailed Description of Each Measure Used in This Trial

**eReferences**

This supplementary material has been provided by the authors to give readers additional information about their work.

© 2022 Cheung AT et al. *JAMA Network Open.*

eTable. The Menu of Strategies of Brief Motivational Interviewing

| Strategies | Aims | Examples of messages |
|---|---|---|
| 1. Opening strategy: lifestyle, stresses, and current level of PA | To build rapport and understand the child's current lifestyle, stresses, and engagement in PA | Would you like to talk about your child's current PA practice in general? What are your child's current stressors? What are your child's current barriers and facilitators? How does your child's current PA practice fit into his/her daily lives? |
| 2. Opening strategy: health and current level of PA | To establish a collaborative relationship and determine the association between the child's health and the current physical activity practice | How does your child's current PA practice affect his/her health? How do you think your child's current PA practice is associated with his/her health? |
| 3. Discussing a typical day | To provide the parents with a comprehensive discussion of their child's current PA practice, and to raise the parents' awareness of the relationship between PA and the events in his/her child's life and assess their readiness to change | Can you tell me what a typical day is for your child from beginning to end? Can you describe a typical day for your child, what happens, how your child felt, and where their current PA practice fits in? |
| 4. The good things and the less good things of engagement in regular physical activity | To enable parents to explore the pros and cons of changing their child's PA level; to identify both the good and less good sides of their ambivalence simultaneously | What are some of the positives and negatives of your child's current PA practice?' How does this affect your child? What do you like and dislike about your child's current PA practice?' What doesn't your child like about it? |
| 5. Providing information | To provide parents with relevant information about the regular exercise practices in a neutral and nonpersonalized manner through a patient-centered approach and assess the parents' readiness to receive information | What connection, if any, do you note between physical inactivity and health? Would you like to know more about how PA can benefit your child's health? I wonder what you would do after knowing…? What do you make of this information? How does this information relate to your current PA practice? |
| 6. Present and future | To create discrepancies by focusing on the child's present and future behavior with the intent to motivate the parents to consider | How would you like things to be different in the future? What is stopping you from doing these things? |

© 2022 Cheung AT et al. *JAMA Network Open.*

| | | |
|---|---|---|
| | encouraging their child to perform regular PA | |
| 7. Exploring concerns | To enable the parents to identify and explore their concerns about their child's engagement in regular PA | What concerns do you have about your child's engagement in regular PA? What other concerns or worries do you have? |
| 8. Helping with decision-making | To assist the parents with making decisions to encourage their child to perform regular PA | What does this information leave you with? What are you planning to do now? What are you going to do now? |

Abbreviation: PA, physical activity.

© 2022 Cheung AT et al. *JAMA Network Open.*

**eAppendix. A Detailed Description of Each Measure Used in This Trial**

The Chinese University of Hong Kong: Physical Activity Rating for Children and Youth (CUHK-PARCY) was used to assess the children's physical activity levels. Survivors were asked to rate their overall weekly levels of physical activity over the past 6 months from 0 (no exercise at all) to 10 (vigorous exercise almost every day), accounting for the frequency, duration, and intensity of the physical activity performed. Scores of 0–2, 3–6, and 7–10 represent low, moderate, and high physical activity levels, respectively. The psychometric properties of this scale have been empirically tested, indicating a good internal consistency with a content validity index of 90% and satisfactory test-retest reliability at a 2-week interval of 0.86 in Hong Kong Chinese children and adolescents.[1]

The Chinese version of the Fatigue Scale - Child was used to assess the survivors' levels of cancer-related fatigue. The survivors were asked to rate their experiences with cancer-related fatigue over the previous 7 days. The scale consists of 13 items, which are evaluated on a 5-point Likert Scale, from 1 = 'not at all' to 5 = 'a lot'. The scores ranged from 13 to 65, with higher scores indicating higher levels of cancer-related fatigue. The psychometric properties of this scale have been tested in a local validation study, yielding a good internal consistency and test-retest reliability and acceptable convergent, discriminant, and known-group validity in 200 Hong Kong Chinese paediatric cancer survivors.[2]

© 2022 Cheung AT et al. *JAMA Network Open.*

This scale has been used in previous local studies examining the levels of cancer-related fatigue in Hong Kong Chinese paediatric cancer survivors.

A hand-held dynamometer was used to measure the children's left- and right-hand grip strengths. Before the measurement, the handle of the hand-held dynamometer was adjusted such that the base could rest on the first metacarpal (heel of the palm) and the handle could rest on the middle of the four fingers. During the test, the children were instructed to push as hard as they could, keeping their arms at right angles and their elbows by their sides. The children were asked to repeat the test three times, with 10 to 20 s of rest between each push. Only the highest of the three measurements for each subject were recorded.[3]

A mini-Wright Standard Handheld peak flow meter was used to measure the children's peak expiratory flow rates. This is a widely available and useful physiological test to assess an individual's maximal expiratory flow rate during a forceful expiration following full inspiration. The participants were asked to take a deep breath and blow into the mouthpiece as fast, powerfully, and fully as possible in a single blow, while ensuring that a tight seal was maintained between the mouthpiece and lip. The steps were repeated two more times and the best of the three blows was recorded. The mini-Wright Standard Handheld peak flow meter was shown to be an accurate and precise device to determine how well an individual's airway works by measuring one's peak expiratory flow rate.[4-6]

© 2022 Cheung AT et al. *JAMA Network Open.*

The survivors' QoL was evaluated using the Chinese version of the Paediatric QoL Inventory 4.0 Generic Core Scales (PedsQL 4.0 generic version), which comprises 23 items rated on a 5-point Likert scale ranging from 0 (never a problem) to 4 (almost always a problem). The items are categorised into four domains as follows: physical functioning (8 items), emotional functioning (5 items), social functioning (5 items), and school functioning (5 items). The score of each item required reverse scoring and linear transformation to a 100-point scale (0–100, 1–75, 2–50, 3–25, and 4–0); thus, the range of possible scores was 0–100, with higher scores representing better QoL. The results of the psychometric properties test showed that it has good internal consistency, with a Cronbach's alpha coefficient of 0.86, and test-retest reliability at 2-week intervals ranging from $r = 0.65$ to $r = 0.81$.[7]

© 2022 Cheung AT et al. *JAMA Network Open.*

**eReferences**


1. Chung OK, Li HC, Chiu SY, Ho KY, Lopez V. The impact of cancer and its treatment on physical activity levels and behavior in Hong Kong Chinese childhood cancer survivors. *Cancer Nurs*. 2014;37(3):E43-E51. doi:10.1097/NCC.0b013e3182980255
2. Ho KY, Li WH, Lam KW, Chiu SY, Chan CF. The Psychometric Properties of the Chinese Version of the Fatigue Scale for Children. *Cancer Nurs*. 2016;39(5):341-348. doi:10.1097/NCC.0000000000000297
3. Hand Grip Strength Protocol. The Center for Drug Abuse and AIDS Research; https://cdaar.tufts.edu/protocols/Handgrip.pdf. Updated 2003. Accessed August 12, 2018.
4. Gupta S, Mittal S, Kumar A, Singh KD. Peak expiratory flow rate of healthy school children living at high altitude. *N Am J Med Sci*. 2013;5(7):422-426. doi:10.4103/1947-2714.115781
5. Jones KP, Mullee MA. Measuring peak expiratory flow in general practice: comparison of mini Wright peak flow meter and turbine spirometer. *BMJ*. 1990;300(6740):1629-1631. doi:10.1136/bmj.300.6740.1629
6. Koyama H, Nishimura K, Ikeda A, Tsukino M, Izumi T. Comparison of four types of portable peak flow meters (Mini-Wright, Assess, Pulmo-graph and Wright Pocket meters). *Respir Med*. 1998;92(3):505-511. doi:10.1016/s0954-6111(98)90299-2


© 2022 Cheung AT et al. *JAMA Network Open.*

7. Chan LF, Chow SM, Lo SK. Preliminary validation of the Chinese version of the Pediatric Quality of Life Inventory. *Int J Rehabil Res*. 2005;28(3):219-227. doi:10.1097/00004356-200509000-00004

© 2022 Cheung AT et al. *JAMA Network Open.*
