# Supplementary material for: Efficacy of Mobile Instant Messaging–Delivered Brief Motivational Interviewing for Parents to Promote Physical Activity in Pediatric Cancer Survivors: A Randomized Clinical Trial
Source: JAMA Netw Open. 2022 Jun 14;5(6):e2214600. doi: 10.1001/jamanetworkopen.2022.14600 (PMC9198728; doi:10.1001/jamanetworkopen.2022.14600)
Supplement: Supplement 3. — Data Sharing Statement [file jamanetwopen-e2214600-s00.pdf]

Data Sharing Statement

Cheung. Efficacy of Mobile Instant Messaging-Delivered Brief Motivational Interviewing for Parents to Promote Physical Activity in Pediatric Cancer Survivors. *JAMA Netw Open*. Published June 14, 2022. doi:10.1001/jamanetworkopen.2022.14600

Data
Data available: Yes
Data types: Deidentified participant data
How to access data: The datasets generated during and/or analyzed during the current study are available from the corresponding author on reasonable request. Email address: williamli@cuhk.edu.hk
When available: With publication

Supporting Documents
Document types: None

Additional Information
Who can access the data: Researchers whose proposed use of the data has been approved
Types of analyses: For a specified purpose
Mechanisms of data availability: After approval of a proposal
